# Supplementary material for: APETx-Like Peptides from the Sea Anemone Heteractis crispa, Diverse in Their Effect on ASIC1a and ASIC3 Ion Channels
Source: Toxins (Basel). 2020 Apr 20;12(4):266. doi: 10.3390/toxins12040266 (PMC7232172; doi:10.3390/toxins12040266)
Supplement: Supplementary file 1 [file toxins-12-00266-s001.pdf]

Supplementary Materials: APETx-Like Peptides from the Sea Anemone *Heteractis crispa*, Diverse in Their Effect on ASIC1a and ASIC3 Ion Channels

Rimma S. Kalina, Sergey G. Koshelev, Elena A. Zelepuga, Natalia Y. Kim, Sergey A. Kozlov, Emma P. Kozlovskaya, Margarita M. Monastyrnaya and Irina N. Gladkikh

Table S1. Hcr 1b-2 intermolecular non-covalent interactions with rASIC1a.

Type[1]	Hcr 1b-2	rASIC1a	Energy contribution, kcal/mol
DIH	Lys40	C-Glu177	−14.18
DH	Lys5	B-Lys341	−9.85
DH	Lys5	B-Glu342	−3.50
DH	Lys41	C-Glu177	−3.00
DH	Lys40	C-Ala178	−2.40
D	His7	C-Asn120	−1.15
D	His7	B-Asn119	−1.14
D	Tyr9	B-Thr236	−0.98
D	Lys41	B-Asp237	−0.66
D	Ile10	B-Pro346	−0.40
D	Ile10	B-Asp349	−0.35
D	His7	B-Thr236	−0.30
D	His7	B-Glu113	−0.25
D	Tyr9	B-Glu235	−0.12
D	Lys41	B-Asp349	−0.09

[1] H – hydrogen bonds, I – ionic, D – distance (hydrophobic, Van der Waals).

Table S2. Hot spots of Hcr 1b-4 intermolecular non-covalent interactions with rASIC1a.

Type[1]	Hcr 1b-4	rASIC1a	Energy contribution, kcal/mol
DIH	Arg41	A-Asp237	−31.61
DIH	Arg41	A-Asp349	−14.30
DH	Arg19	A-Asp126	−9.47
DI	Lys40	A-Asp237	−5.01
DH	Tyr9	A-Asp345	−4.30
DH	Tyr7	A-Glu342	−2.68
DH	Gly23	B-Cys172	−2.15
DH	Asn27	A-Glu235	−1.22
DH	Tyr24	B-Leu169	−1.13
D	Leu28	A-Ala116	−1.01
D	Leu28	A-Thr236	−1.01
DH	Ser22	A-Glu235	−0.90
DA	Lys40	B-His173	−0.90

[1] H - hydrogen bonds, I - ionic, D - distance (hydrophobic, Van der Waals).

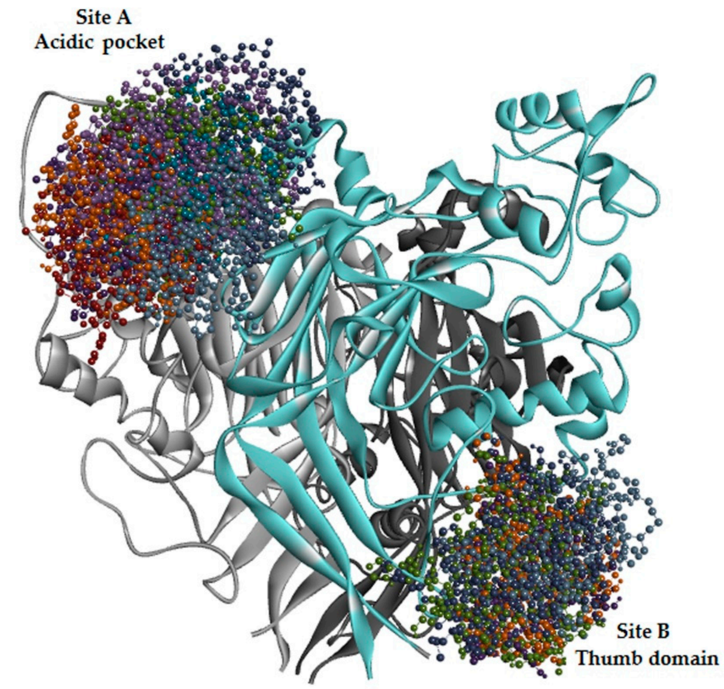
Site A
Acidic pocket
Site B
Thumb domain

(a)

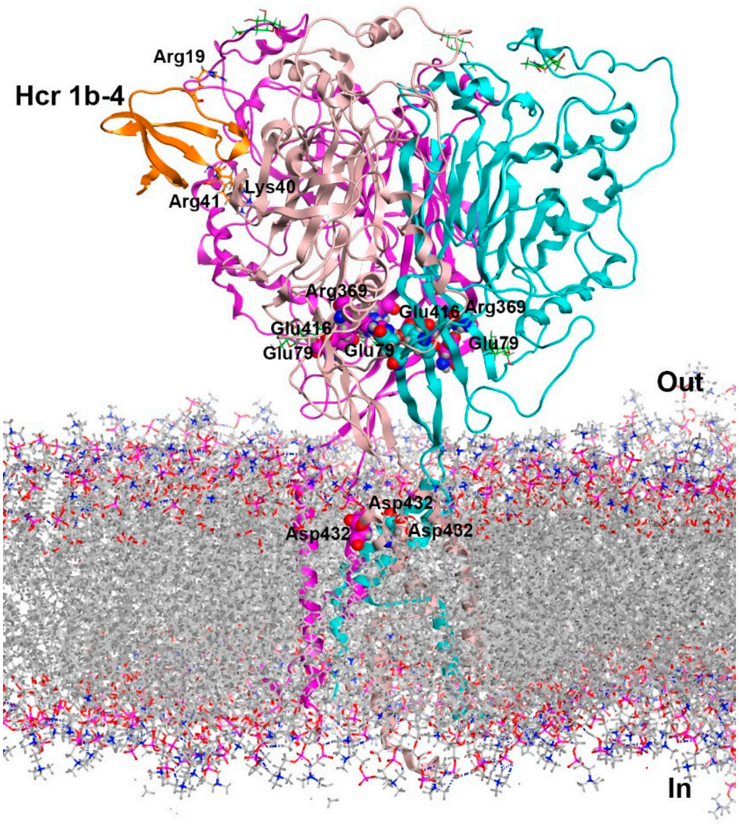
Hcr 1b-4
Arg19
Lys40
Arg41
Arg369
Glu416
Glu79
Glu79
Glu416
Arg369
Glu79
Out
Asp432
Asp432
Asp432
In

(b)

Figure S1. 3D structure models of Hcr 1b-2 (**a**) and Hcr 1b-4 (**b**) complexes with rASIC1a. (**a**) Two possible sites of Hcr 1b-2 interaction on rASIC1a trimer predicted by ClusPro2.0. The ribbon representation of the rASIC1a extracellular region (three subunits are colored blue, grey and dark grey) and ball and stick representation of 10 top docked clusters Hcr 1b-2 at site A (population from 33 to 253 and the lowest energy score –861.1) and site B (population from 32 to 81 and the lowest energy score –232.6). (**b**) Ribbon diagram of the Hcr 1b-4–rASIC1 complex embedded into DPPC lipid bilayer (represented as gray sticks). The Arg19, Lys40 and Arg41 side chains of Hcr 1b-4 are represented as bold sticks, and the channel residues involved in a circle pattern of inter- and intra-subunit hydrogen bonds and ionic interactions within the ASIC1a central vestibule as well as pore residues of Asp432 are represented as balls and labeled. Part of the DPPC lipid bilayer as well as solvent molecules have been removed for clarity. Visualization was performed with Discovery studio 4.0 Visualizer software [41] (**a**) and MOE 19.0102 [42] (**b**).
